# The Role of microRNAs in Epigenetic Regulation of Signaling Pathways in Neurological Pathologies

**DOI:** 10.3390/ijms241612899

**Published:** 2023-08-17

**Authors:** Pavel P. Tregub, Irada Ibrahimli, Anton S. Averchuk, Alla B. Salmina, Peter F. Litvitskiy, Zaripat Sh. Manasova, Inga A. Popova

**Affiliations:** 1 Department of Pathophysiology, I.M. Sechenov First Moscow State Medical University, 119991 Moscow, Russia; 2 Scientific and Educational Resource Center “Innovative Technologies of Immunophenotyping, Digital Spatial Profiling and Ultrastructural Analysis”, RUDN University, 117198 Moscow, Russia; 3 Research Center of Neurology, 125367 Moscow, Russia; 4 Research Institute of Molecular Medicine and Pathobiochemistry, Prof. V. F. Voino-Yasenetsky Krasnoyarsk State Medical University, 660022 Krasnoyarsk, Russia

**Keywords:** miRNA, Alzheimer’s disease, ischemia–reperfusion injury, blood–brain barrier, RNA biology, neuroinflammation, oxidative stress

## Abstract

In recent times, there has been a significant increase in researchers’ interest in the functions of microRNAs and the role of these molecules in the pathogenesis of many multifactorial diseases. This is related to the diagnostic and prognostic potential of microRNA expression levels as well as the prospects of using it in personalized targeted therapy. This review of the literature analyzes existing scientific data on the involvement of microRNAs in the molecular and cellular mechanisms underlying the development of pathologies such as Alzheimer’s disease, cerebral ischemia and reperfusion injury, and dysfunction of the blood–brain barrier.

## 1. Introduction

Discovered 30 years ago, microRNAs (miRNAs) are small non-coding RNA molecules that are 18–25 nucleotides long (22 nucleotides on average) and involved in the transcriptional and post-transcriptional regulation of gene expression by RNA interference, which is of great interest to molecular biologists, geneticists, and biochemists [1,2]. These molecules are mainly present intracellularly, but there is also an extracellular (circulating) microRNA fraction [3]. To date, the existence and functions of more than 2500 human miRNAs are known [4]. The existing database of fundamental knowledge about the structure and functional significance of microRNAs has significantly increased researchers’ interest in studying the role of these molecules in oncological and hereditary pathologies [5]. Promising trends include the diagnostic and prognostic value of assessing microRNA expression levels and their use as targets for personalized targeted therapy [6].

The determination of the role of microRNAs in the epigenetic regulation of signaling pathways in the pathogenesis of multifactorial disorders such as Alzheimer’s disease, ischemia–reperfusion injury, and dysfunction of the blood–brain barrier deserves special attention [7,8,9]. The aim of this review is to summarize and analyze the results of contemporary scientific data on the involvement of microRNAs in the molecular and cellular mechanisms underlying the development of these neurological pathologies.

## 2. Role of miRNAs in Epigenetic Regulation of Genome Translation

Approximately 70% of the human genome is transcribed into non-coding RNAs that serve regulatory functions. Among them are ribosomal RNA, transfer RNA, small nuclear RNA, and small nucleolar RNA. Non-coding RNAs also include small interfering RNAs, microRNAs, and long non-coding RNAs, which influence the normal expression of target genes, making them a novel target for the development of targeted therapy.

Small interfering RNAs (siRNAs) regulate endonucleolytic cleavage of mRNA and participate in the epigenetic regulation of translation through the RNA interference mechanism, leading to a natural “knockdown” of the target gene [1,10]. MicroRNAs can suppress the expression of target genes and inhibit the translation process. They are capable of recognizing specific mRNA sequences, even with only 6–8 nucleotides at the 5’-end. Long non-coding RNAs control genome integrity, chromatin organization, gene expression, and signal transmission. It has been established and experimentally confirmed that the targeted action of non-coding RNAs on mRNA (in the form of complementary antisense oligonucleotides) can alter the expression levels of target genes [11].

MicroRNAs are efficient signaling regulators of genomic translation. They can bind to several different targets on mRNA, and multiple microRNA variants can be specific to one mRNA target [1]. Additionally, the involvement of microRNAs in processes of positive regulation of transcription and translation has been demonstrated [12]. Such an epigenetic mechanism is undoubtedly phylogenetically more recent and characteristic of higher eukaryotes [3].

The majority of genes encoding microRNAs are antisense and transcribed as independent units [13], but sometimes microRNAs are located in a sense orientation and are co-transcribed with the gene that encodes their mRNA target locus [14]. Nearly half of the known microRNAs are generated from polycistronic units and are encoded by genes located within the introns of non-coding genes [15]. Additionally, for 16% of microRNAs, RNA editing has been demonstrated, allowing for the generation of different microRNA variants from one gene [16].

Mature microRNAs become part of the active complex RISC/microRNP (RNA-induced silencing complex/microRNA–ribonucleoprotein complex) [17]. Proteins of the Argonaute (Ago) family and chaperones Hsp70/Hsp90 play a central role in assembling RISC [18,19]. Once RISC is assembled, Ago proteins are essential for mRNA interference, and they possess two conserved domains for binding to microRNAs: the PAZ domain (binding the 3’-end) and the PIWI domain (binding the 5’-end) [20]. The mature microRNA within the RISC complex is oriented to interact with the target mRNA region [21].

**Figure 1:**
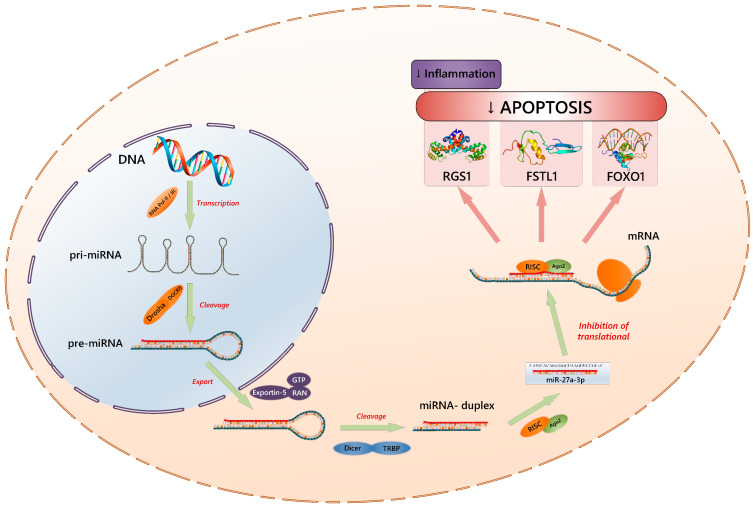
The process of RNA interference involving microRNAs in the example of miR-27a-3p. DNA—deoxyribonucleic acid; RNA Pol II/III—RNA polymerase II or III; pri-miRNA—primary miRNA; Drosha—class 2 ribonuclease III enzyme; DGCR8—DiGeorge syndrome critical region 8; pre-miRNA—precursor miRNA; RAN—RAs-related nuclear protein; GTP—guanosine triphosphate; Dicer—endoribonuclease Dicer or helicase with RNase motif; TRBP—transactivation response RNA binding protein; RISC—RNA-induced silencing complex; Ago2—Argonaute 2; mRNA—messenger RNA; RGS1—regulator of G protein signaling 1; FSTL1—follistatin-related protein 1; FOXO1—forkhead box protein O1, ↓—inhibition.

Suppression of gene expression can occur by degrading the mRNA or preventing its translation. If the microRNA is fully complementary to the target mRNA, Ago2 cleaves the mRNA and leads to its degradation [22]. In the case of partial complementarity, RNA interference is achieved through translation blocking (Figure 1).

## 3. Methods for Assessing the Pool and miRNA Expression

For a detailed investigation of the role of microRNAs in physiological and pathological processes, several methods for isolating microRNAs have been developed, but their stability often raises concerns [23,24]. MicroRNA molecules degrade much more easily than mRNA, which is due to their short length and the activity of RNases [13]. Consequently, all steps involving sample handling require materials, solutions, and tools that are thoroughly RNase-free, and the investigated samples may need good cooling or even additional stabilization using nanomaterials [25].

One of the standard and widely used methods for detecting microRNAs is Northern blotting [26,27]. It can be used to detect not only mature microRNAs but also their precursors. For quantitative assessment of microRNA expression (both absolute and relative), polymerase chain reaction (PCR) methods can be utilized: reverse transcription PCR followed by amplification and real-time fluorescent detection [28,29], isothermal loop-mediated amplification [30], and rolling circle amplification [31,32].

Biosensor [33] and microarray [34] methods are known for hybridizing microRNAs, enabling the simultaneous detection of a large number (hundreds or thousands) of microRNA targets. High-throughput sequencing (Sanger sequencing or next-generation sequencing) [35] is used to identify new microRNA sequences.

To study spatiotemporal expression and intracellular transport, it is essential to investigate microRNA expression in vivo, for which the in situ hybridization method is most suitable [36]. For specific detection of microRNAs in situ, locked nucleic acid (LNA) probes [37] or morpholinos [38] can be used. LNA, due to its locked conformation, exhibits high sensitivity and specificity for detecting microRNA molecules [37]. Moreover, through using LNA and morpholinos, the activity level of microRNAs can be experimentally inhibited [39]. Similar potential exists with 2′-O-methylated oligoribonucleotides and complementary oligonucleotides called antagomirs [40]. Additionally, microRNA maturation and their binding to mRNA sites can be arrested at various points using space-blocking oligonucleotides [41].

One of the modern and promising methods for assessing microRNA effects is the technology of multiplex detection based on microsphere arrays [36,42,43]. This technology enables high-throughput detection of both proteins and nucleic acid targets in various samples. Xu Y et al. [44] described the development of a set of fluorophore-labeled microspheres (known as rainbowarray microspheres). The authors also modified the liquid hybridization method for multiplex detection of microRNA targets and demonstrated the practicality of the technology by quantitatively measuring the expression of two types of microRNA during the differentiation of 3T3-L1 cells.

Methods for measuring the levels of circulating microRNAs in blood serum deserve special attention as they can serve as diagnostic biomarkers for various diseases [45,46,47,48,49,50,51]. The most commonly used methods for this purpose include quantitative reverse transcription polymerase chain reaction (qRT-PCR) [52], loop-mediated isothermal amplification [53,54], and other amplification techniques [55]. There are reports of one-step direct measurement of microRNA molecules in human blood serum using a modified beacon probe with LNA and fluorescence spectroscopy and microscopy [56] as well as reports of amplification with magnetic nanoparticles and ligation [57].

To enhance the efficiency of microRNA detection methods in blood serum, which hold significant diagnostic prospects, approaches utilizing nanomaterials [58] are becoming popular. For instance, superparamagnetic nanoparticles coated with polyethylene glycol and biotin conjugates [59] or intracellular gold nanoprobes [60] are being employed for this purpose.

Performing precise quantitative determination of microRNA pools is methodologically challenging and often associated with a high number of errors [55]. Therefore, bioinformatic methods are crucial for studying microRNA expression levels and investigating their effects at this level [61]. Comparison of data on mRNA targets and microRNAs (based on sequence information) is carried out in specialized databases [62,63]. Sophisticated bioinformatic methods and analytical tools have been developed for comparing data on mRNA targets and microRNAs [64,65,66,67].

## 4. The Role of MicroRNAs in Signaling Pathways of Alzheimer’s-Type Neurodegeneration

Disruption of epigenetic regulation and alterations in microRNA expression, which usually occur in conjunction, are important factors in the pathogenesis of many neurological disorders [8,9].

Several recent and extensive systematic reviews [68,69,70,71,72,73] have addressed the role of microRNAs in the pathogenesis of Alzheimer’s disease. These reviews explore numerous microRNA variants involved in the epigenetic regulation of the neurodegenerative process that serve as potential targets for diagnosis and targeted therapy.

Examples of such microRNAs include the molecules miR-200b, miR-135a, miR-10a-5p, miR-142a-5p, miR-146a-5p, miR-155-5p, miR-211-5p, miR-455-5p, miR-34a, miR-125b, miR-181c, miR-9, miR-191-5p, miR-181c, and miR-206, which are considered potential diagnostic markers for Alzheimer’s disease [74]. Additionally, attention should be paid to microRNA molecules that possess both diagnostic and therapeutic potential: miR-128, miR-574, miR-146-a, miR-181, miR-132, miR-188-5p, and miR-137 [74].

There are also studies describing modern bioinformatic approaches that use artificial intelligence and machine learning algorithms for identifying new biomarkers and improving the accuracy of molecular diagnosis of Alzheimer’s disease [75,76,77,78,79].

Some review papers present important data on other non-coding RNAs, such as circular RNAs and long non-coding RNAs, which participate in the pathogenesis of neurodegeneration and regulate the interplay between microRNAs/mRNAs/regulatory signaling pathways, which are mediated genetically [80,81]. These RNA molecules are often considered by researchers as more efficient targets for diagnostic and therapeutic strategies. Another important contemporary research direction in Alzheimer’s disease pathogenesis is the study of the regulatory functions of both microRNAs localized in specific organelles (such as mitochondria [82]) and exosomal microRNAs [83,84].

The assessment of neurovascular dysfunction is of particular interest as it plays a crucial role in the onset and progression of the neurodegenerative process associated with the accumulation of β-amyloid peptide in brain neurons and cerebral vessel walls [85,86]. Special attention is given to the dysfunction of the blood–brain barrier, reduced cerebral blood flow, and impaired vascular clearance of beta-amyloid from the brain into the glymphatic system and meningeal lymphatic vessels [87]. These disturbances may, in turn, be linked to the reprogramming of epigenetic regulation [88,89].

According to several authors, RNA interference mediated by microRNAs can initiate neurovascular events leading to Alzheimer’s-type neurodegeneration [90,91,92,93]. This provides a strong theoretical basis for the development of new directions in targeted genetic therapy for neurodegenerative diseases [94,95,96,97].

## 5. Regulation of Signaling Pathways in Ischemia and Reperfusion Injury of Nerve Cells Involving microRNAs

In in vitro models, it has been shown that the activation of miR-496 and miR-874-3p reduces the consequences of ischemic–reperfusion injury in neurons by negatively regulating BCL2L14 and BMF/BCL2L13, respectively [98,99]. Activation of miR-92b-3p expression inhibits apoptosis, mitochondrial dysfunction, and inflammation through the inhibition of TRAF3 [100]. MiR-182-5p and miR-193b-3p also exert neuroprotective effects in cerebral ischemia–reperfusion injury by negatively regulating the inflammatory response mediated by Toll-like receptor 4 and 5-lipoxygenase, respectively [101,102]. Additionally, miR-30c acts by inhibiting neuronal apoptosis in ischemia–reperfusion injury of the brain, thus suppressing the expression of SOX9/MAPK [103,104], while miR-449a downregulates the expression of amphiregulin (AREG) [105].

Similar effects are observed with the inhibition of miR-19a-3p, which reduces the extent and area of cerebral ischemia–reperfusion injury by regulating inflammation and apoptosis through increased expression of IGFBP3 both in vivo and in vitro [106]. It has also been discovered that inhibiting exosomal miR-200a-3p/141-3p, which originates from astrocytes and targets the gene SIRT1 and its associated signaling pathways, reduces the pathological consequences of cerebral ischemia–reperfusion injury in a mouse model [107]. MiR-370 has been shown to exacerbate neuronal reperfusion injury by impacting the expression of SIRT6 and the regulation mechanism of the Nrf2/ARE signaling pathway [108], while the exosomal form of this microRNA (370-3p) increases blood–brain barrier permeability during ischemia–reperfusion injury through the interference of MAPK1 [109].

Long non-coding RNA MEG3, by binding to miR-485, promotes the exacerbation of cerebral ischemia–reperfusion injury through potentiating pyroptosis via AIM2 [110]. Additionally, the knockdown of miR-155-5p acts by inhibiting the pyroptosis mechanism and inflammation through interference with DUSP14 [111,112]. It should be noted that Zhang L and colleagues [113], without emphasizing the orientation of miR-155, also demonstrated its activating influence on apoptosis and inflammatory processes in neural tissue.

**Figure 2:**
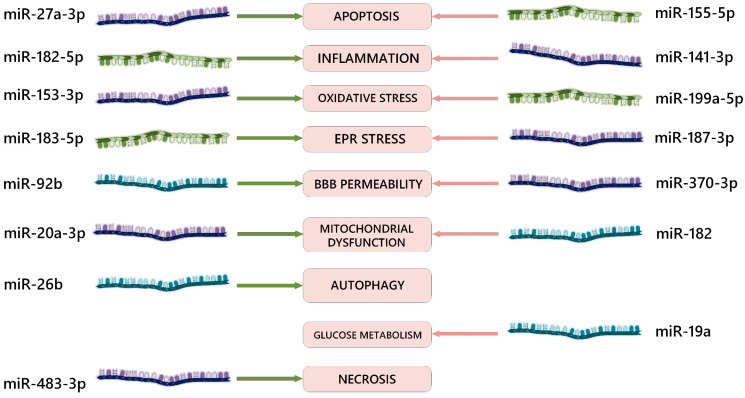
Examples of effects of various miRNAs on signaling pathways in the pathogenesis of ischemia–reperfusion brain injury. EPR = endoplasmic reticulum; BBB = blood–brain barrier; green arrows = positive biological effect of RNA interference; red arrows = negative biological effect of RNA interference. The dark blue color of the molecules indicates microRNA-3p (sense); the olive color indicates microRNA-5p (antisense); the aquamarine color indicates microRNAs with an unknown formation end from pre-microRNA.

In addition to the mentioned studies, there are several other works concerning the role of microRNAs in the pathogenesis of ischemia–reperfusion injury and the signaling pathways through which their effects are realized during RNA interference (Table 1, Figure 2). The majority of neuroprotective mechanisms affected by the overexpression or inhibition of microRNAs involve anti-apoptotic, anti-inflammatory, and antioxidant signaling pathways.

Most studies from various researchers have demonstrated unidirectional effectiveness regarding the neuroprotective role of miR-10b-3p [116,117], miR-124-3p [119,120], miR-141-3p [107,131], miR-153-3p [141,142], miR-182-5p [101,150], miR-20a-3p [162,163], miR-22-3p [171,172], miR-24-3p [175,176], miR-27a-3p [181,182,183], miR-342-5p [191,192], miR-455-3p [200,201], miR-488-3p [203,204], and miR-92b-3p [100,221] as well as the damaging role of miR-141-3p [107,131], miR-155-5p [111,112], and miR-182 [148,149]. Therefore, these specific microRNAs should be considered as priority targets for further translation into clinical practice.

Indeed, along with the discovery of unidirectional effects of microRNA expression, contradictory results concerning the same molecules are also encountered. For example, according to the findings presented by Jia T et al. [205], activation of miR-489-3p expression in in vivo and in vitro models reduces the pathological consequences of cerebral ischemia–reperfusion by inhibiting histone deacetylase 2 (HDAC2), thereby reducing apoptosis intensity and enhancing cell viability. In contrast, Song L et al. [206] obtained opposing results: in mice subjected to temporary middle cerebral artery occlusion, an intensification of oxidative stress and neuron apoptosis was observed under conditions of increased miR-489-3p levels, which inhibits Sirtuin1 (SIRT1).

In the study investigating the effects of miR-421-3p in cerebral ischemia–reperfusion [199], both in vivo and in vitro models showed that increased expression of miR-421-3p reduces the intensity of inflammation through the YTHDF1/NF-κB p65 signaling pathway. On the contrary, Xu J. et al. [198] found a reverse effect of this microRNA concerning the intensity of apoptosis and inflammation in models of ischemia–reperfusion nerve tissue damage, mediated through the myocyte enhancer factor 2C (MEF2C).

In the study by Wei XY et al. [170], positive effects of the long non-coding RNA (lncRNA) RPL34-AS1 were observed in patients with stroke as well as in cerebral ischemia in rats and in an OGD cell model. This molecule inhibits miR-223-3p, which targets the insulin-like growth factor 1 receptor (IGF1R). The authors attribute the positive effects of RPL34-AS1 to its influence on this specific mechanism. However, there are contradictory results regarding the effects of miR-223-3p in studies focusing on the circular RNA circPDS5B and its impact on angiogenesis [168] and regarding the positive impact of miR-223-3p expression on the development of the inflammatory response in the zone of ischemia–reperfusion and its surrounding area [169].

The evaluation of the effects of miR-19a and its sense form miR-19a-3p shows a negative role in the mechanism of cerebral ischemia–reperfusion injury played by excessive stimulation of apoptosis and inflammation [106,156,157]. Similar observations are confirmed for the structurally related miR-19b-3p, which also intensifies the neuroinflammatory process in the ischemia–reperfusion zone [157]. However, these observations are contradicted by data showing that knockdown of the long non-coding RNA H19 and overexpression of miR-19a-3p attenuated the severity of ischemia–reperfusion-induced oxidative stress and apoptosis in neurons, as reported by Gao N. et al. [158].

The study of the effects of the lncRNA Malat1 revealed a positive influence of miR-26b [179] and a negative impact of miR-142-3p expression [132] on apoptosis and cell proliferation during experimental brain hypoxia–ischemia. However, the results from Li J. et al. [133] show an opposite effect when miR-142-3p expression is enhanced: inhibiting FBXO3. It is important to note that this study has limitations as it was conducted only on an in vitro model.

In two studies investigating the role of miR-140-3p on in vitro models with OGD, opposite effects were demonstrated: on the PC12 cell line, an enhancement of miR-140-3p expression showed a weakening effect on apoptosis and oxidative stress [130], while on the N2a cell line, an overexpression of miR-140-3p potentiated apoptosis and oxidative stress [129]. Supporting the greater significance of the first study and the neuroprotective role of this molecule is the fact that the in vitro results obtained by Zhang Y. et al. were replicated in the study on patients with ischemic stroke.

Another contradiction in determining the role of miRNAs in the pathogenesis of ischemia–reperfusion brain injury is the data on miR-128-3p. They indicate its proapoptotic efficacy in in vitro and in vivo models by inhibiting the FOXO/Relaxin signaling pathway [122] as well as the neuroprotective efficacy (potentiation of differentiation and myelination processes) of this miRNA in exosomal localization in an in vivo experiment [123].

Moreover, the studies mentioned above demonstrate that miR-135a, miR-181c, and miR-211-5p, whose expression plays a positive role in protection against cerebral ischemia–reperfusion injury, also act as neuroprotective agents in Alzheimer’s disease [74]. Conversely, the overexpression of miR-155-5p, miR-200a-3p, and miR-206 leads to damage to neural tissue both in the context of ischemia–reperfusion and Alzheimer’s-like neurodegeneration [74].

## 6. Peculiarities of Epigenetic Regulation in BBB Dysfunction

Modern data underscore the important role of blood–brain barrier (BBB) dysfunction in the development of various neurological pathologies [222]. Disruption of the integrity and permeability of the BBB is a significant element in the pathogenesis of hypoxic–ischemic and infectious brain injuries [223,224,225]. There is also evidence showing specific features of BBB functioning in developmental brain disorders and in autoimmune and neurodegenerative pathologies [226,227,228,229]. 

Increased permeability of the blood–brain barrier (BBB) in conditions of chronic brain hypoxia–ischemia during the peri- and neonatal periods may be one of the contributing factors to the progression of neurodegenerative processes. This can occur due to the entry of barrier-breaking antigens into the peripheral bloodstream, leading to subsequent immune responses and dysfunction of proteolytic and neurotransmitter systems [230]. There is compelling experimental evidence of a disrupted tight junction structure and dysregulation of BBB transporter proteins in Alzheimer’s disease and Parkinson’s disease [228,231].

Epigenetic factors responsible for DNA methylation and histone remodeling are crucial for normal ontogenetic development of the brain and its barrier structures [88,232]. Among these factors are small non-coding RNA molecules [233]. They are susceptible to reprogramming under the influence of hypoxic stimuli, which can result in both damage and preconditioning effects [234,235]. If the stimulus is excessive and/or prolonged, it can lead to the development of hypoxic–ischemic brain injury and contribute to future neurological disorders.

It has been shown that blocking histone deacetylase in microglia culture exerts a protective effect on oligodendrocytes after experimental hypoxia–ischemia [236]. Moreover, the M1 fraction suppresses the activity of oligodendrocytes, while the M2 fraction enhances it and allows for reducing the expression of pro-inflammatory factors and increasing the expression of anti-inflammatory cytokines. Methylation and demethylation processes involve a large number of enzymes, including histone acetyltransferases, deacetylases, histone methyltransferases, and demethylases [237]. These processes contribute to the modulation of transcription in response to endogenous and exogenous factors such as hypoxia and inflammation [238]. In in vivo models, it has been demonstrated that experimental hypoxia–ischemia in the neonatal period leads to increased expression of caspase-3, decreased expression of synapsin, and inhibition of histone methylation H3K4me2/-me3 and H3K27me2/-me3, resulting in the induction of neuronal apoptosis in the hippocampus [239].

Hypoxia–ischemia disrupts the structure and organization of the components of the blood–brain barrier (BBB), leading to increased permeability of the cerebral endothelium [240]. Degradation of BBB structures has been shown to increase the risk of early stroke, while blocking BBB disruption provides protection to the brain parenchyma [241]. The degradation of tight junction proteins or cell adhesion proteins under the influence of metalloproteinases is directly regulated by microRNAs [242]. Specifically, miR-539, miR-132, miR-21, and miR-671-5p inhibit the expression of MMP-9 and protect endothelial cells during experimental ischemia, preventing increased BBB permeability [243,244,245,246]. Bai Y and colleagues [247] found that circular RNA DLGAP4 positively regulates endothelial–mesenchymal transition, associated with BBB integrity, through miR-143 during ischemic stroke. Interestingly, the positive effect in the form of apoptosis suppression upon the inhibition of miR-143 expression is also observed in the ischemia–reperfusion injury model [135]. Increased adhesion of immune cells to the damaged endothelium contributes to their infiltration into surrounding tissue. Certain microRNAs can help preserve BBB integrity by reducing immune cell adhesion and the expression of pro-inflammatory cytokines [235]. For example, miR-210 activates the expression of TNF-α, IL-1β, IL-6, and chemokine ligands CCL1 and CCL2, which are linked to the pro-inflammatory response in a mouse model of ischemic stroke [248]. 

It is worth noting that miR-210 is recognized as one of the key regulators of neonatal hypoxia–ischemia [249]; its overexpression, along with that of miR-130a, increases BBB permeability by inhibiting the expression of occludin and β-catenin [250,251]. Overexpression of miR-126-3p/-5p and miR-98 in the ischemic brain of mice suppresses the effects of pro-inflammatory cytokines and adhesion molecules, preserving the integrity of the cerebral endothelium and thus reducing the negative consequences of stroke [252,253,254]. A similar effect is observed with overexpression of miR-152-3p, which reduces the degree of neurological deficit and disruption of BBB integrity in experimental ischemia by inhibiting apoptosis of endothelial cells through negative modulation of the MAP3K2/JNK/c-Jun pathway [255].

On the contrary, miR-34a is activated in endothelial cells during episodes of acute hypoxia–ischemia, which negatively affects mitochondrial function in these cells by targeting cytochrome C [256]. Moreover, knockout of miR-34a reduces BBB permeability, attenuates disruptions of tight intercellular contacts, improves stroke outcomes [242], and improves cognitive functions in mice with neurodegeneration [74].

MiR-15a is also activated in the cerebral endothelium of mice after acute oxygen–glucose deprivation, leading to cell death. This process is inhibited when the δ-receptor is activated by the peroxisome proliferator-activated receptor (PPAR), which acts as a potential neuroprotective factor during ischemic stroke [257]. The main role in maintaining BBB permeability belongs to the endothelium and, to a lesser extent, pericytes. It has been shown that during hypoxic–ischemic injury, pericytes detach from the basal membrane, contributing to increased BBB permeability. This process is induced by miR-149-5p [258].

There is also evidence of the involvement of other microRNAs in regulating the structure and functions of the cerebral endothelium. For instance, the upregulation of miR-150-5p leads to the negative regulation of the Vezf1 protein synthesis, resulting in dysfunction of the endothelial cells in the cerebral cortex’s blood vessels [259].

Particular attention should be given to the molecule miR-132, the expression of which not only exerts a protective effect on the blood–brain barrier [244] but also reduces oxidative stress and autophagy during cerebral ischemia–reperfusion injury [124] and possesses neuroprotective properties in Alzheimer’s disease [74].

## 7. Conclusions

The materials reviewed in this study indicate the significant importance of epigenetic regulation, including the involvement of microRNAs, in the pathogenesis of blood–brain barrier dysfunction, neurodegenerative disorders, and ischemia–reperfusion injuries of the nervous tissue. This has generated increasing interest in advancing technologies for studying the properties of these molecules and evaluating the prospects for translating this knowledge into practical medicine for predicting neurological pathologies, their differential diagnosis, and the search for targets for targeted therapies.

## Figures and Tables

**Table 1:** Effects of various miRNAs on target proteins and signaling pathways in the pathogenesis of ischemia–reperfusion brain injury.

microRNA	Target Protein/Signaling Pathway	Direction of Gene Expression	Ischemia/OGD Effect	Ref.	Biological Effect of RNA Interference
miR-101-3p	↓ HDAC9	↑	↓ apoptosis ↑ morphology of neurons	[114]	Positive
miR-101a-3p	↑ Dusp1	↓	↓ apoptosis	[115]	Negative
miR-10b-3p	↓ PDCD5	↑	↓ apoptosis	[116]	Positive
	↓ KLF5	↑	↓ apoptosis ↓ swelling ↓ inflammation	[117]	Positive
miR-122-5p	↓ SEMA3A	↑	↓ apoptosis ↓ oxidative stress	[118]	Positive
miR-124-3p	↓ Nrep/Rnf38	↑	↓ apoptosis	[119]	Positive
	↓ CTDSP1/AKT	↑	↓ apoptosis ↓ axon damage	[120]	Positive
miR-1247-3p	↓ Caspase-2	↑	↓ apoptosis	[121]	Positive
miR-128-3p *	↑ FOXO/Relaxin	↓	↓ apoptosis	[122]	Negative
	↓ ACVR1/BMP	↑	↓ demyelination ↑ differentiation	[123]	Positive
miR-132-3p	↓ ATG12 ↑ p-PI3K/p-AKT/mTOR	↑	↓ oxidative stress ↓ autophagy	[124]	Positive
miR-133a-3p	↓ DAPK1 ↑ p-Akt и p-mTOR	↑	↓ apoptosis ↓ autophagy	[125]	Positive
miR-133b	↓ TRAF3/NF-κB	↑	↓ apoptosis	[126]	Positive
miR-134	↓ HSPA12B	↓	↓ apoptosis	[127]	Negative
miR-135a-5p	↓ NR3C2	↑	↓ apoptosis ↓ oxidative stress ↓ inflammation	[128]	Positive
miR-140-3p *	↑ HIF-1α	↑	↓ apoptosis ↓ oxidative stress ↓ inflammation	[129]	Positive
	↓ Tyro3/PI3K/Akt ↓ Bax/Caspase-3 ↑ Bcl-2	↑	↑ apoptosis ↑ oxidative stress	[130]	Negative
miR-141-3p	↑ SIRT1/ZO-1/Occludin/Claudin-5/CD31 ↓ p-NF-κB/IL-1β/TNF-α/GFAP	↓	↓ inflammation	[107]	Negative
	↑ PBX1/PROK2	↓	↓ apoptosis	[131]	Negative
miR-142-3p *	↑ SIRT1/SOD/Catalase ↓ TNF-α/IL-6/IL-1β/ROS/MDA	↓	↓ apoptosis	[132]	Negative
	↓ FBXO3	↑	↓ apoptosis ↓ inflammation	[133]	Positive
miR-142-5p	↑ Nrf2/ARE	↓	↓ oxidative stress	[134]	Negative
miR-143-3p	↑ FSTL1 ↓ Bax/Caspase-3 ↑ Bcl-2	↓	↓ apoptosis	[135]	Negative
miR-144-3p	↑ Brg1/Nrf2/ARE	↓	↓ apoptosis ↓ oxidative stress	[136]	Negative
miR-145	↓ FOXO1	↑	↓ apoptosis ↓ oxidative stress ↓ inflammation	[137]	Positive
miR-148b-3p	↑ Sestrin2/Nrf2	↓	↓ apoptosis ↓ oxidative stress	[138]	Negative
miR-149-5p	↓ Notch2	↑	↓ apoptosis ↓ inflammation	[139]	Positive
miR-152-3p	↓ PSD-93 ↑ Nrf2/ARE	↑	↓ apoptosis ↓ oxidative stress	[140]	Positive
miR-153-3p	↓ SRC/MAPK	↑	↓ apoptosis ↓ oxidative stress ↓ inflammation	[141]	Positive
	↓ FOXO3	↑	↓ apoptosis	[142]	Positive
miR-153-5p	↓ TLR4/p65/IkBa	↑	↓ apoptosis	[143]	Positive
miR-155	↑ MafB ↓ IL-1β/IL-6/TNF-α ↓ iNOS/COX-2	↓	↓ apoptosis ↓ inflammation	[113]	Negative
miR-155-5p	↑ DUSP14/TXNIP/NLRP3	↓	↓ inflammation ↓ pyroptosis	[112]	Negative
	↑ DUSP14/NF-kB/MAPKs	↓	↓ apoptosis	[111]	Negative
miR-181a	↑ PTEN	↓	↓ apoptosis ↓ oxidative stress	[144]	Negative
miR-181a-5p	↑ En2/Wnt/β-catenin	↓	↓ apoptosis	[145]	Negative
miR-181c-3p	↓ CXCL1	↑	↓ inflammation	[146]	Positive
miR-181d	↑ DOCK4	↓	↓ apoptosis ↓ inflammation	[147]	Negative
miR-182	↑ mTOR/FOXO1/Bcl-2/Bax	↓	↓ apoptosis	[148]	Negative
	↑ cortactin	↓	↓ mitochondrial dysfunction ↓ inflammation	[149]	Negative
miR-182-5p	↓ TLR4	↑	↓ inflammation	[101]	Positive
	↓ Rac1	↑	↓ inflammation	[150]	Positive
miR-186-5p	↑ MDM4	↓	↓ apoptosis ↓ oxidative stress	[151]	Negative
miR-187-3p	↓ GRP78/Seipin	↑	↑ apoptosis ↑ endoplasmic reticulum stress	[152]	Negative
miR-193b	↓ ATG7	↑	↓ autophagy ↓ ferroptosis	[153]	Positive
miR-193b-3p	↓ 5-lipoxigenase	↑	↓ inflammation	[102]	Positive
miR-199a-5p	↑ Brg1/Nrf2/HO-1	↓	↓ apoptosis ↓ oxidative stress	[154]	Negative
miR-199b	↓ AQP4	↑	↓ oxidative stress ↓ inflammation	[155]	Positive
miR-19a	↑ ADIPOR2	↓	↓ apoptosis ↑ glucose metabolism	[156]	Negative
miR-19a-3p *	↑ FOXO3/SPHK1/NF-kB p65 ↓ SIRT1	↑	↑ inflammation	[157]	Negative
	↑ IGFBP3	↓	↓ apoptosis ↓ inflammation	[106]	Negative
	↓ PTEN/PI3K/AKT	↑	↓ apoptosis ↓ oxidative stress	[158]	Positive
miR-19b-3p	↑ FOXO3/SPHK1/NF-kB p65 ↓ SIRT1	↑	↑ inflammation	[157]	Negative
miR-200a-3p	↑ SIRT1/ZO-1/Occludin/Claudin-5/CD31 ↓ p-NF-κB/IL-1β/TNF-α/GFAP	↓	↓ inflammation	[107]	Negative
miR-200b-3p	↑ β-TrCP	↓	↓ apoptosis	[159]	Negative
miR-202-5p	↓ eIF4E/ ↑ Akt/GSK-3β	↑	↓ apoptosis ↓ autophagy	[160]	Positive
miR-203a-3p	↓ SRC/MAPK	↑	↓ apoptosis ↓ oxidative stress ↓ inflammation	[141]	Positive
miR-206	↑ USP22/Sirt1	↓	↓ apoptosis ↓ inflammation	[161]	Negative
miR-20a-3p	-	↑	↓ cognitive dysfunction	[162]	Positive
	↓ MMP, IL-17A	↑	↓ mitochondrial dysfunction ↓ inflammation	[163]	Positive
miR-21-3p	↑ CAMKK2/AMPK/Nrf-2	↓	↓ oxidative stress ↓ inflammation	[164]	Negative
miR-211-5p	↓ COX2 ↓ PGD2/PGE2/TNF-α/IL-1β	↑	↓ apoptosis ↓ inflammation	[165]	Positive
miR-216a	↓ JAK2/STAT3 ↓ iNOS и MMP-9/TNF-α и IL-1β	↑	↓ apoptosis ↓ inflammation	[166]	Positive
miR-22	↑ Wnt/β-catenin and PKC/ERK	↑	↓ apoptosis	[167]	Positive
miR-223-3p *	↓ NOTCH2	↑	↑ angiogenesis	[168]	Positive
	↓ CysLT 2 R	↑	↓ inflammation	[169]	Positive
	↑ IGF1R	↓	↓ apoptosis ↑ glucose metabolism	[170]	Negative
miR-22-3p	↓ KDM6B/BMP2/BMF	↑	↓ apoptosis	[171]	Positive
	↓ IL-1β, IL-18/Caspase-1	↑	↓ pyroptosis ↓ inflammation	[172]	Positive
miR-224-3p	↓ FIP200	↑	↓ apoptosis	[173]	Positive
miR-23a-3p	↓ CXCL12	↑	↓ apoptosis	[174]	Positive
miR-24-3p	↑ NRP1/NF-κB p65	↓	↑ apoptosis ↑ inflammation	[175]	Positive
	↓ BOK	↑	↓ apoptosis ↓ oxidative stress	[176]	Positive
miR-25-3p	↓ TRAF3	↑	↓ apoptosis ↓ inflammation	[177]	Positive
miR-26a-5p	↓ DAPK1	↑	↓ apoptosis	[178]	Positive
miR-26b	↑ ULK2	↓	↑ autophagy	[179]	Positive
miR-26b-5p	↓ KLF10/N-myc/PTEN	↑	↓ apoptosis ↓ inflammation	[180]	Positive
miR-27a-3p	↓ FOXO1/p27 Kip1	↑	↓ apoptosis	[181]	Positive
	↓ FSTL1	↑	↓ apoptosis	[182]	Positive
	↓ Rgs1	↑	↓ apoptosis ↓ inflammation	[183]	Positive
miR-29a-3p	↓ NF-κB	↑	↓ apoptosis	[184]	Positive
miR-302b-3p	↑ FGF15/Nrf2/ARE	↓	↓ apoptosis ↓ oxidative stress	[185]	Negative
miR-30a-5p	↑ YWHAG	↓	↓ apoptosis ↓ oxidative stress ↓ inflammation	[186]	Negative
miR-30c	↓ SOX9/MAPK	↑	↓ apoptosis	[103]	Positive
miR-30c-5p	↑ Rock2/MAPK	↓	↓ apoptosis	[104]	Negative
miR-32-3p	↓ Cab39/AMPK	↑	↑ apoptosis ↑ oxidative stress	[187]	Negative
miR-325-3p	↓ RIP3	↑	↓ apoptosis	[188]	Positive
miR-32-5p	↓ PTEN/PI3K/AKT	↑	↓ cell necrosis	[189]	Positive
miR-337-3p	↑ YBX1	↓	↓ apoptosis	[190]	Negative
miR-342-5p	↑ PFN1	↓	↑ apoptosis	[191]	Positive
	CCAR2/Akt/NF-κB	↑	↓ apoptosis	[192]	Positive
miR-361-3p	↑ NACC1/PINK1/Parkin	↑	↓ apoptosis ↓ oxidative stress	[193]	Positive
miR-363-3p	↓ DUSP5	↑	↓ cell necrosis	[194]	Positive
miR-370	↓ SIRT6/Nrf2/ARE	↑	↓ apoptosis ↓ oxidative stress ↓ inflammation	[108]	Positive
miR-370-3p	↓ MAPK1	↑	↑ BBB permeability	[109]	Negative
miR-372-3p	↓ TLR4	↑	↓ apoptosis	[195]	Positive
miR-383-5p	↓ HDAC9	↑	↓ apoptosis ↓ endoplasmic reticulum stress	[196]	Positive
miR-410	↓ FOXO3	↑	↓ apoptosis ↓ oxidative stress	[197]	Positive
miR-421-3p *	↑ MEF2C	↓	↓ apoptosis	[198]	Negative
	↓ YTHDF1/NF-κB p65	↑	↓ inflammation	[199]	Positive
miR-449a	↓ AREG/EGFR/PI3K/Akt	↑	↓ apoptosis	[105]	Positive
miR-455-3p	↓ PDCD7	↑	↓ apoptosis	[200]	Positive
	↓ TP53INP1	↑	↓ apoptosis ↓ oxidative stress ↓ inflammation	[201]	Positive
miR-485	↑ AIM2	↓	↑ pyroptosis	[110]	Positive
miR-485-5p	↓ Rac1/Notch2	↑	↓ apoptosis ↓ inflammation	[202]	Positive
miR-488-3p	↓ RAC1	↑	↓ apoptosis ↓ inflammation	[203]	Positive
	↓ VPS4B	↑	↓ cell necrosis	[204]	Positive
miR-489-3p *	↓ HDAC2	↑	↓ apoptosis	[205]	Positive
	↓ SIRT1	↑	↑ apoptosis ↑ oxidative stress	[206]	Negative
miR-494-3p	↓ Bhlhe40	↑	↑ apoptosis ↑ oxidative stress	[207]	Negative
miR-496	↓ BCL2L14	↑	↓ apoptosis	[98]	Positive
miR-497	↑ bcl-2/bcl-w	↓	↓ apoptosis	[208]	Negative
miR-499a-5p	↓ PDCD4	↑	↓ apoptosis	[209]	Positive
miR-520a-3p	↓ IRF9	↑	↓ apoptosis	[210]	Positive
miR-532-5p	↓ CXCL1/CXCR2/NF-κB	↑	↓ apoptosis ↓ oxidative stress ↓ inflammation	[211]	Positive
miR-613	↓ ATG3	↑	↓ apoptosis	[212]	Positive
miR-652-3p	↑ Bcl-2 ↓ Bax	↑	↓ apoptosis	[213]	Positive
miR-665-3p	↓ TRIM8/NF-κB	↑	↓ apoptosis ↓ inflammation	[214]	Positive
miR-666-3p	↓ MAPK1	↑	↓ apoptosis	[215]	Positive
miR-7-5p	↑ RelA p65	↓	↓ apoptosis ↓ oxidative stress ↓ inflammation	[216]	Negative
miR-874	↓ BMF/BCL2L13	↑	↓ apoptosis	[99]	Positive
miR-874-3p	↓ ATG16L1	↑	↓ apoptosis	[217]	Positive
miR-92b	↑ NOX4	↓	↑ BBB damage	[218]	Positive
miR-92b-3p	↓ TRAF3	↑	↓ apoptosis ↓ mitochondrial dysfunction ↓ inflammation	[100]	Positive
	↓ NOX4	↑	↓ apoptosis ↓ oxidative stress ↓ inflammation	[100]	Positive
miR-9-5p	↑ FOXO/Relaxin	↓	↓ apoptosis	[122]	Negative
miR-98-5p	↓ BCL2L13	↑	↓ apoptosis ↓ oxidative stress ↓ inflammation ↓ endoplasmic reticulum stress	[219]	Positive
miR-99b	↓ IGF1R	↑	↓ apoptosis	[220]	Positive

Note: *—conflicting data from different studies. ↓—inhibition; ↑—induction; ACVR1—Activin A receptor type I; ADIPOR2—Adiponectin receptor 2; AIM2—absent in melanoma 2; AKT—alpha serine/threonine-protein kinase; AMPK—Adenosine monophosphate-activated protein kinase; AQP4—Aquaporin-4; ARE—antioxidant response element; AREG—amphiregulin; ATG16L1—autophagy-related 16 like 1; ATG3/7/12—autophagic conjugate 3/7/12; Bax—BCL2-associated X protein; BBB—blood–brain barrier; bcl-2—B-cell lymphoma 2; BCL2L13/14—B-cell lymphoma 2-like protein 13/14; bcl-w—anti-apoptotic member of the BCL-2 family of proteins; Bhlhe40—basic helix–loop–helix family member e40; BMF—bone marrow failure; BMP—basic metabolic panel; BOK—Bcl-2-related ovarian killer; Brg1—brahma-related gene-1; Cab39—calcium-binding protein 39; CAMKK2—calcium/calmodulin-dependent protein kinase 2; CCAR2—cell cycle and apoptosis regulator 2; CD—cluster of differentiation; COX-2—Cyclooxygenase-2; CTDSP1—C-terminal domain small phosphatase 1; CXCL1/2/12—chemokine (C–X–C motif) ligand 1/2/12; CysLT 2 R—Cysteinyl leukotriene receptor 2; DAPK1—death-associated protein kinase 1; DOCK4—dedicator for cytokinesis 4; DUSP1/5/14—dual specificity phosphatase 1/5/14; EGFR—epidermal growth factor receptor; eIF4E—eukaryotic translation initiation factor 4E; En2—Engrailed-2; ERK—extracellular signal-regulated kinase; FBXO3—F-Box protein 3; FGF15—fibroblast growth factor 15; FIP200—family-interacting protein of 200 kDa; FOXO1/3—forkhead box transcription factors 1/3; FSTL1—follistatin-like 1; FSTL1—follistatin-related protein 1; GFAP—glial fibrillary acidic protein; GRP78—Glucose-regulated protein 78; GSK-3β—Glycogen synthase kinase-3 beta; HDAC2—Histone Deacetylase 2; HDAC9—Histone Deacetylase 9; HIF-1α—hypoxia-inducible factor 1-alpha; HO-1—Heme oxygenase-1; HSPA12B—heat shock protein A12B; IGF1R—insulin-like growth factor 1 receptor; IGFBP3—insulin-like growth factor binding protein 3; IkBa—nuclear factor of kappa light polypeptide gene enhancer in B-cells inhibitor, alpha; IL-1β/6/17A/18—interleukin-1β/6/17A/18; iNOS—inducible nitric oxide synthase; IRF9—interferon regulatory factor 9; JAK2—Janus Kinase 2; KDM6B—Lysine demethylase 6B; KLF5/10—Krueppel-like factor 5/10; MafB—V-maf musculoaponeurotic fibrosarcoma oncogene homolog B; MAPK—mitogen-activated protein kinase; MDA—maternally-derived antibodies; MDM4—Murine double minute 4; MEF2C—myocyte-specific enhancer factor 2C; MMP-9- Matrix metalloproteinase 9; mTOR—mammalian target of rapamycin; NACC1—nucleus accumbens-associated 1; NF-κB—nuclear factor kappa B; NLRP3—nucleotide-binding domain, leucine-rich family, pyrin domain-containing-3; N-myc—N-myc proto-oncogene; NOTCH2—neurogenic locus notch homolog protein 2; NOX4—NADPH oxidase 4; NR3C2—nuclear receptor subfamily 3, group C, member 2; Nrep—neuronal protein; Nrf2—nuclear factor erythroid 2–related factor 2; NRP1—neuropilin-1; p27 Kip1—Cyclin-dependent kinase inhibitor 1B; PBX1—pre-B-cell leukemia transcription factor 1; PDCD4/5/7—programmed cell death 4/5/7; PFN1—Profilin 1; PGD2/E2—Prostaglandin D2/E2; PINK1—PTEN-induced putative kinase 1; PKC—Protein Kinase C; p-PI3K—Phosphoinositide 3-kinases; PROK2—Prokineticin 2; PSD-93—post-synaptic density 93; PTEN—Phosphatase and Tensin homolog; Rac1—Ras-related C3 botulinum toxin substrate 1; RelA p65—v-rel avian reticuloendotheliosis viral oncogene homolog A; Rgs1—regulator of G protein signaling 1; RIP3—receptor-interacting Serine/Threonine-protein Kinase 3; Rnf38—RING finger protein 38; Rock2—Rho-associated coil-containing protein kinase 2; ROS—reactive oxygen species; SEMA3A—Semaphorin3a; SIRT1/6—Sirtuin 1/6; SOD—Sphincter of Oddi Dysfunction; SOX9—SRY-Box transcription factor 9; SPHK1—sphingosine kinase 1; SRC—non-receptor tyrosine kinases; STAT3—signal transducer and activator of transcription 3; TLR4—Toll-like receptor 4; TNF-α—tumor necrosis factor alpha; TP53INP1—tumor protein 53-induced nuclear protein 1; TRAF3—tumor necrosis factor receptor-associated factor 3; TRIM8—Tripartite motif-containing 8; TXNIP—Thioredoxin-interacting protein; Tyro3—Tyrosine-protein kinase receptor 3-member; ULK2—Unc-51-like autophagy-activating Kinase 2; USP22—Ubiquitin-specific Peptidase 22; VPS4B—Vacuolar protein-sorting 4B; Wnt—Wingless-related integration site; YBX1—Y-Box binding protein 1; YTHDF1—YTH domain family, member 1; YWHAG—Tryptophan 5-Monooxygenase activation protein gamma; ZO-1—zonula occludens 1; β-TrCP—beta-Transducin repeat-containing E3 Ubiquitin protein Ligase.

## Data Availability

Not applicable.

## Abbreviations

AIM2	Interferon-inducible protein AIM2 (absent in melanoma 2)
AREG	Amphiregulin
BCL2L14	Apoptosis facilitator Bcl-2-like protein 14 is a protein that, in humans, is encoded by the BCL2L14 gene
BCL2L13	A protein which, in humans, is encoded by the BCL2L13 gene on chromosome 22
BBB	Blood–brain barrier
CCL1	Chemokine (C-C motif) ligand 1
DNA	Deoxyribonucleic acid
DLGAP4	Disks large-associated protein 4 (DAP-4)
DUSP14	Dual-specificity Phosphatase 14
FOXO	Forkhead box protein O1 (FOXO1), also known as forkhead in rhabdomyosarcoma (FKHR)
FBXO3	F-box protein 3
GTP	Guanosine triphosphate
HDAC2	Histone deacetylase 2
HSP70	The 70-kilodalton heat shock proteins
HSP90	The 90-kilodalton heat shock proteins
Il-1β	Interleukin-1 beta
Il-6	Interleukin 6
IGF1R	Insulin-like growth factor 1 receptor
IGFBP3	Insulin-like growth factor-binding protein 3
LNA	Locked nucleic acid
MAPK 1	Mitogen-activated protein kinase 1
MEG3	Maternally expressed 3 imprinted long non-coding RNA gene
MEF2C	Myocyte enhancer factor 2C
MMP9	Matrix metallopeptidase 9
MAP3K2	Mitogen-activated protein Kinase 2
mRNA	Messenger RNA
Nrf2/ARE	Nuclear factor erythroid 2-related factor 2
OGD	Oxygen–glucose deprivation
PCR	Polymerase chain reaction
qRT-PCR	Quantitative reverse transcription polymerase chain reaction
RNA	Ribonucleic acid
RPL34-AS1	Ribosomal protein L34
RISC/microRNP	RNA-induced silencing complex/miR–ribonucleoprotein complex
RNase	Ribonuclease
SOX9	SRY (sex-determining region Y)-Box
SIRT1	(Silent mating type information regulation 2 homolog) 1
SIRT6	Stress-responsive protein deacetylase and mono-ADP ribosyl transferase enzyme encoded by the SIRT6 gene
siRNAs	Small interfering RNAs
TRAF3	TNF receptor-associated factor
TRBP	Transactivation response RNA binding protein
TNF-α	Tumor necrosis factor
YTHDF1	YTH domain family; member 1 is a protein that, in humans, is encoded by the YTHDF1 gene
